# High rates of COVID-19 vaccine refusal among Afghan pregnant women: a cross sectional study

**DOI:** 10.1038/s41598-022-18497-x

**Published:** 2022-08-18

**Authors:** Arash Nemat, Sumaira Yaftali, Tamim Jan Danishmand, Haroon Nemat, Nahid Raufi, Abdullah Asady

**Affiliations:** 1 Department of Microbiology, Kabul University of Medical Sciences, 3rd District, Kabul, 1001 Afghanistan; 2 Department of Cardiology, Ariana Medical Complex, Kabul, Afghanistan; 3 Department of Gynecology, Kabul University of Medical Sciences, Kabul, Afghanistan; 4 Department Surveillance, Ministry of Public Health, Kabul, Afghanistan; 5 Department of Pediatrics, Rahmat Medical Complex, Kabul, Afghanistan; 6 Department of Dermatology, Maiwand Hospital, Kabul University of Medical Sciences, Kabul, Afghanistan

**Keywords:** Health care, Medical research

## Abstract

Severe Acute Respiratory Syndrome Corona Virus Type-2 (SARS-COV-2) was first detected in China and created a global pandemic rapidly. Subsequently after serious efforts different types of vaccines produced against the virus and recommended for all people including pregnant women. The aim of this study was to realize the willingness of pregnant women to accept the COVID-19 vaccine in Kabul Afghanistan. For this purpose, a cross-sectional study was conducted in gynecology wards of several hospitals in Kabul, Afghanistan from 10th of July to 20th of August 2021 through direct interview with the pregnant women who had come for prenatal care to the healthcare centers. The collected data were analyzed through Statistical Package for Social Studies (SPSS) version 25. Simple descriptive analysis computed for demographic characteristics and bi-variable (Chi-square) analysis was carried out to find out the associations of taking vaccine with demographic variables. A p-value of < 0.05 was considered significant at 95% confidence interval. A total of 491 who were completed the inclusion criteria included in the analysis. A small portion of pregnant women (8.6%) illustrated the intent to get the COVID-19 vaccine if it is recommended by the health authorities in Afghanistan. Our study found a high rate of COVID-19 vaccine refusal among pregnant women in Kabul, Afghanistan. They showed the concern on adverse effects of the vaccine as the main reason for refusal, emphasizing the need to reduce the misconception on vaccine efficacy and campaigns to enhance awareness on the vaccine safety and benefits for mothers and babies.

## Introduction

### Rationale

The Corona Virus Disease-2019 (COVID-19) was first emerged in Wuhan, China^1^. Soon after emergence, it has created a global pandemic spread to almost every corner of the world^2^. Until October 10, 2021, there have been 237,196,253 confirmed cases of the COVID-19, including 4,840,189 deaths worldwide^3^. In Afghanistan, more than 151,000 people tested positive with almost 7000 deaths reported officially^4^.

According to the CDC, pregnant women are considered a vulnerable group for severe illness of the COVID-19 compared to non-pregnant, additionally, they are at increased risk of preterm birth as compared to those not infected with the COVID-19^5^. At the beginning of the COVID-19 vaccination program, many young women did not participate in the clinical trials and declared their concerns over fertility issues and hesitated to get the vaccine^6^.

Currently, the COVID-19 vaccination is recommended for all people, including those who are pregnant, trying to get pregnant, or might become pregnant in the future^5,7^. Studies have not reported any difference in vaccine adverse effects between pregnant and non-pregnant women^8^.

Globally, several reports demonstrated the hesitancy to get COVID-19 vaccine during pregnancy. Insufficient perception about the disease threat, doubts on vaccine safety and benefits and inadequate recommendations from vaccine providers, considered as main obstacles among pregnant women^6,9,10^. Getting vaccinated for both pregnant and non-pregnant considered as an essential step to prevent severe illness, hospitalization and death, particularly in countries with low care facilities for pregnant women and their babies. Furthermore, no report has declared any severe adverse effects of the COVID-19 vaccine on mothers and their fetuses. Therefore pregnant women who have not received any shot of the COVID-19 vaccine should get vaccinated as soon as possible and continue using masks while the vaccine becomes available.

Vaccine refusal refers to delay in the acceptance or hesitancy to vaccination despite availability of vaccines. It is classified as one of the top ten public health issues by WHO in 2019^11^. In other words, it is defined as a set of beliefs, attitudes, or behaviours, or a combination of them, shared by a large and heterogeneous section of a population including people who exhibit reluctant conformism, and vaccine-specific behaviours^12^. Factors such as complacency, convenience and confidence are reported to have influence on vaccine refusal^13^. Based on these factors the development of each vaccine particularly the COVID-19 vaccines directly depend on the availability and acceptance of the vaccine in the community^14^.

Considering the above, this study was carried out to find out refusal rates of COVID-19 vaccine among pregnant women in Kabul, Afghanistan as an important and vulnerable group of society. The findings would help policy makers tailor their plans to address the needs of the pregnant women and the society at large.

### Women’s health in Afghanistan

Afghan women face many common health obstacles. Due to longtime exposure to conflict-related trauma, intimate partner violence and poverty, they suffer from depressive symptoms, food insecurity, gender inequitably, economic dependency and minimal power of decision making in relationship with their husbands^15–17^.

Afghanistan has one of the worst maternal and infant mortality rates globally, according to the World Health Organization (WHO), with 638 women dying per 100,000 live births^18^. This particular group faces plenty of health challenges. A hospital cross sectional study conducted in 2019 reported that more than half of pregnant women (51%) had anemia, indicating a severe public health problem in the country^19^. In addition, the COVID-19 pandemic has also added to their challenges since its emergence. Under the shadow of the COVID-19 pandemic an estimated 116 million babies will be born worldwide, of which, approximately one million will be born in Afghanistan. Virtually the pandemic putting the pregnant women and their babies at a greater risk of infection particularly those who are living in the developing countries like Afghanistan^20^.

At present, women do not have access to most basic health information, family planning ideas, modern methods of contraception, prenatal and postnatal specialty care, and the diagnosis and treatment of cancer and infertility^21^. Health facilities often lack sufficient staffing, essential supplies and equipment. Afghanistan has 4.6 medical doctors, nurses, and midwives per 10,000 population, far below the threshold of 23 healthcare professionals per 10,000 people as defined by WHO^22^. Afghan citizens including women often struggle to access care due to costs, including transportation to a health facility, and medications for which patients are obliged to pay^23^.

As of 31 May 2022, over 6 million COVID-19 vaccine doses were administered in Afghanistan, which were mainly donated from different countries^24^. However, the majority of people still remains unvaccinated. The vaccine is recommended for general population with high-risk group but has not been recommended for pregnant women thus far. Among those who received the vaccines there are no any gender inequity reports. Based on a survey, less than two-thirds of the Afghan public showed their willingness to take the COVID-19 vaccine with a significant portion having reservations to take the vaccine^25^.

Although Afghanistan is looking for vaccine donations from other countries and distribute to the people without costs. As of August 2021 the health authority of Afghanistan does not have COVID-19 vaccine recommendation for pregnant women meanwhile it might be important to realize their willingness for the COVID-19 vaccine acceptance. Subsequently it will inform the health organization and public health policy makers to create a suitable strategy for pregnant vaccination around the country. Therefore, our study aims to define the actual picture of the COVID-19 vaccine acceptance and hesitancy in a sample of pregnant women in Afghanistan.

## Materials and methods

### Study population and data collection

A paper-based cross-sectional study was conducted in gynecology wards of several hospitals in Kabul, Afghanistan to assess the COVID-19 vaccine acceptance and refusal among pregnant women who had come for prenatal care. In this study, the questionnaire was available from 10 July to 20 August 2021 through direct interview with gynecologists who visited the pregnant women. The interviews were conducted in Dari and Pashtu, the national languages of Afghanistan. The goal of study was explained and the items were clearly described to the potential participants. Written consent forms were distributed and for those who were illiterate it was explained verbally. The data were collected and analyzed anonymously without patient’s personal identifications.

### Inclusion and exclusion criteria

All women who were pregnant during the interview and visited the gynecology ward for care were included in this study. Pregnant women who were not from Kabul province and those who had other diseases which might affect their communication were excluded.

### Variables

The questionnaire composed of a brief introductory paragraph which clearly explained the purpose of the study, a declaration on the voluntary, anonymous and confidential nature of the study, with a mandatory informed consent obtained from all participants.

Data were collected on sociodemographic characteristics (i.e., age, occupation, residence, economic status, education level of the participant and education level of her spouse), medical history (i.e., gravidity, parity, and trimester time), source of information, and the history of COVID-19 infection in her or any other family members. Participants indicated their willingness to accept a COVID-19 vaccine by response to question ‘If a vaccine becomes available for COVID-19, would you take it?’ with a Yes or No. If a participant indicated (Yes) option, the interview would end, but if someone selected the negative option (No), the questionnaire would further ask for reasons of refusal to get the vaccine. In this part multiple-choice questions were provided to assess their opinion. These items were highlighted as the main reasons of vaccine refusal in previously published papers. “It leads to infertility”, “It’s not safe for infant”, “It will kill me within two years of time”, “It have magnetic memories”, “I do not need any vaccine because I have enough immunity”, “It might be of lower quality for Afghanistan”, and “other reasons”. The same items were included in our questionnaire.

### Statistical analysis

The data were collected and entered into a Microsoft Excel spreadsheet and then entered into the SPSS version 25 for analysis. Simple descriptive analysis was computed for demographic characteristics, the values for age, gravidity, parity and gestational trimester were explored by mean with standard deviation, and the rest of items were explained in frequency and percentage. Acceptance and refusal to get the vaccine were measured. The reasons for hesitancy to take the COVID-19 vaccine, are presented using a separate table. Bi-variable (Chi-square) analysis was carried out to find out associations of willingness and refusal to take the COVID-19 vaccine with age groups, residency, occupation, economic state, education level, education level of her husband, gestational trimester, gravidity and parity. A p-value of < 0.05 was considered significant at 95% confidence interval.

### Ethical statement

The study approved by the Ethics Committee of Microbiology department of Kabul University of Medical Sciences (KUMS-MD-109). All processes were performed in accordance with relevant guidelines and regulations, including the declaration of Helsinki and subsequent revisions.

### Consent to participate

All study participants provided their informed consents to participate in the study before completing the survey.

### Consent for publication

All contributing authors provided their consent for publication.

## Results

A total of 513 pregnant women participated in the study. From these, 22 women had other health problems, and therefore excluded from analysis. A total of 491 complete sets of the questionnaire were included in the final analysis. The median age of participants was 27 years with maximum 42 and minimum 15 years old. Almost two-thirds of the pregnant women (62.5%; 307/491) were house wives, less than one-third were illiterate (28.9%; 142/491) and almost one quarter of the study participants lived in rural areas (24.8%; 122/491).

About 3 in 4 participants (72.7%; 357/491) indicated busted economic status. Meanwhile a significant proportion of them (16.1%: 79/491) responded that their spouses were illiterates. Slightly less than two-thirds of pregnant women were Multigravida (61.5%; 302/491) and Multiparous (63.3%; 311/491). More than half of the participants (55.2%; 271/491) were in the third trimesters of their pregnancy, as presented in Table 1.

**Table 1:** Overview of the sociodemographic characteristics of the participants (N = 491).

Characteristics	Value
Age	27.24 ± 5.698
Gravidity	1.62 ± 0.487
Parity	1.63 ± 0.482
Gestational trimester	2.36 ± 0.786
**Occupation**	
House wife	307 (62.5%)
Employee	184 (37.5%)
**Residence**	
Urban	369 (75.2%)
Rural	122 (24.8%)
**Level of education**	
Literate	349 (71.1%)
Illiterate	142 (28.9%)
**Husband’s education level**	
Literate	412 (83.9%)
Illiterate	79 (16.1%)
**Economic level**	
Busted	357 (72.7%)
Good	134 (27.3%)
Gravidity*	
Primigravida	189 (38.5%)
Multigravida	302 (61.5%)
Parity**	
Nulliparous	180 (36.7%)
Multiparous	311 (63.3%)
**Gestational trimester**	
First trimester	95 (19.3%)
Second trimester	125 (25.5%)
Third trimester	271 (55.2%)

Values are given as mean ± standard deviation or as number (percentage).

*Gravidity was defined as the sum of all pregnancies, including all live births and pregnancies that terminated at < 6 months or did not result in a live birth.

**Parity was defined as pregnancies that resulted in the delivery at ≥ 6 months gestation, of either a live birth or a stillbirth.

**Figure 1:**
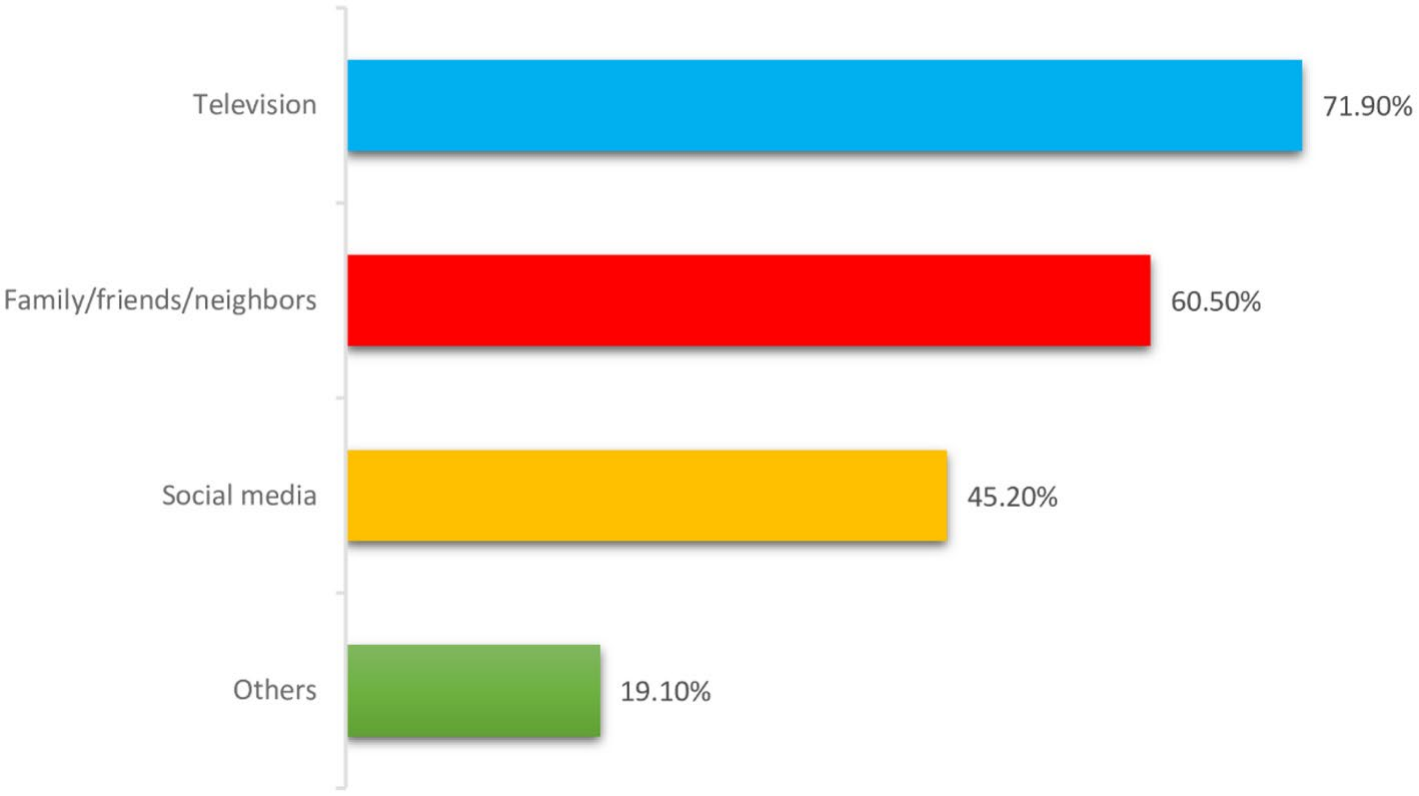
Sources of information used by pregnant women about COVID-19.

Figure 1 shows the information sources of the study participants. Almost 70% of the subjects reported that they got information through television, 60.5% through their families, friends and neighbors, 45.2% through social media, and 19.1% through other resources.

**Figure 2:**
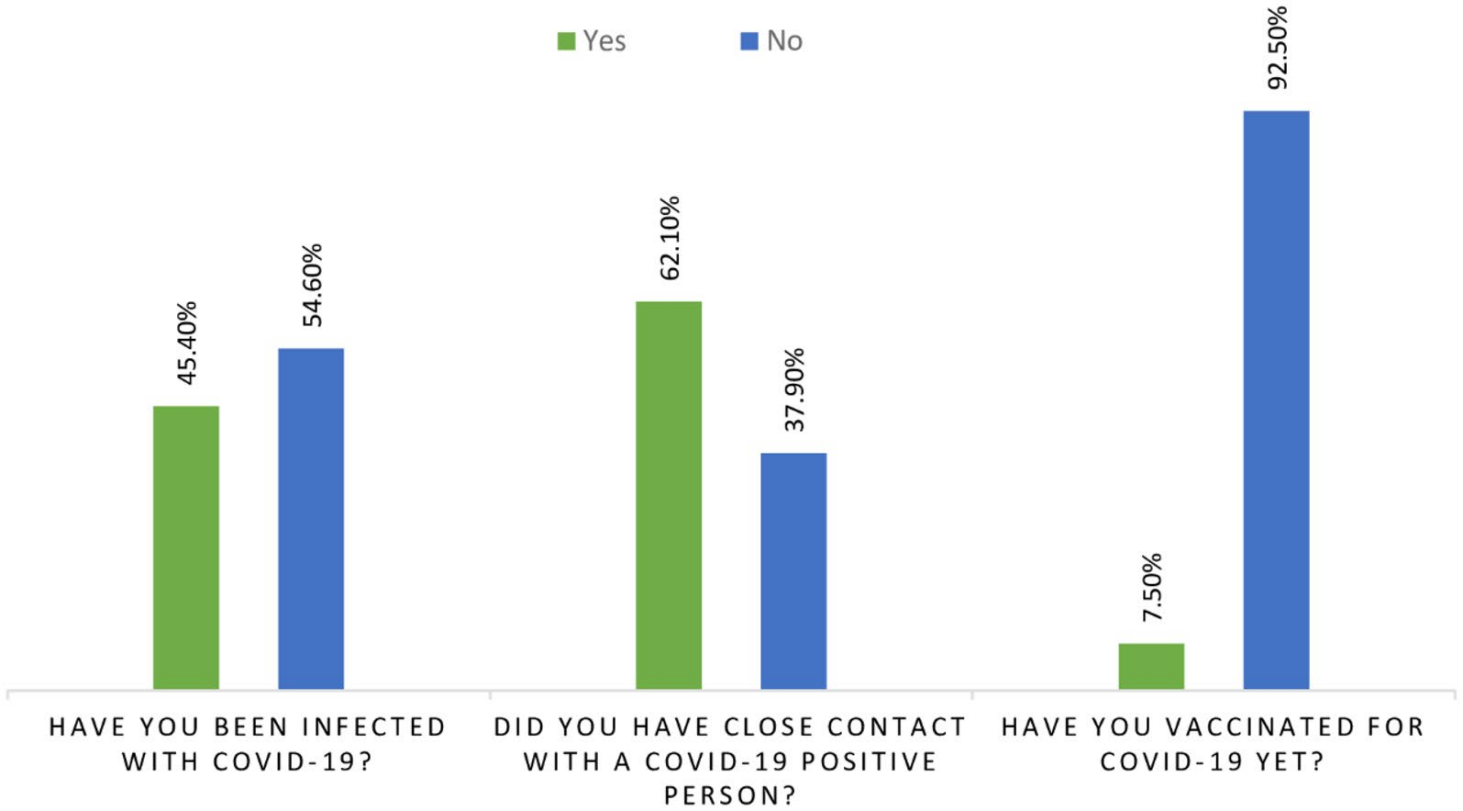
Participants’ declaration on COVID-19 infection state, their proximity contact with infected patients and the vaccination status of participants.

Data on the status of COVID-19 infection among pregnant women, their close contact with infected patients and the vaccination status are presented in Fig. 2. Less than half (45.5%; 223/491) of pregnant women confirmed that they were infected by COVID-19. Almost two-thirds (62.1%; 305/491) reported to have close contacts with COVID-19 patients inside their family or friends.

**Figure 3:**
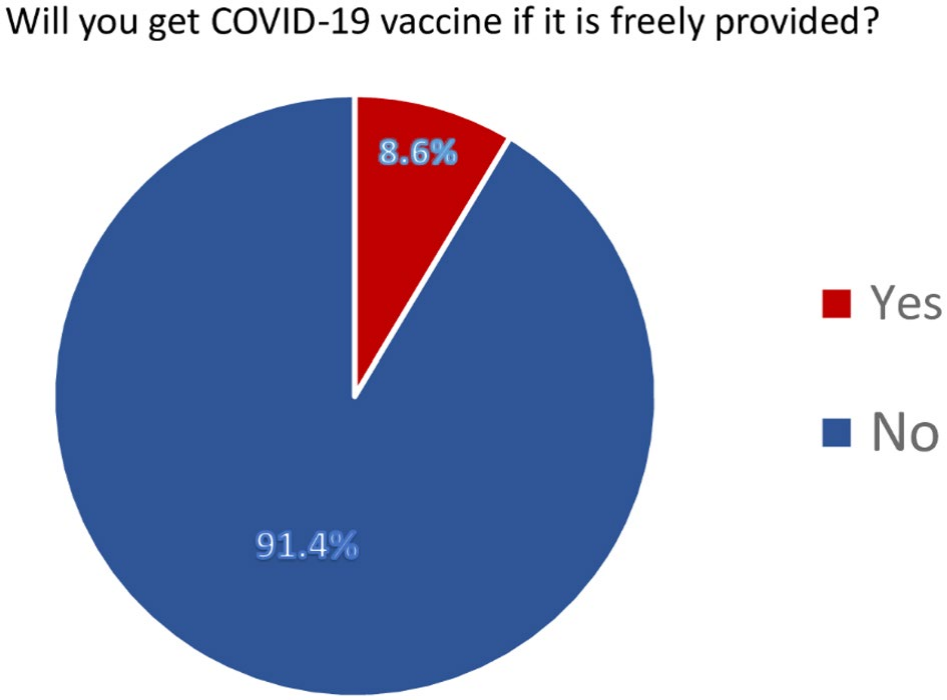
A high proportion of participants declared their refusal to get the COVID-19 vaccine.

Responses to questions on willingness and hesitancy to take the COVID-19 vaccine are illustrated in Fig. 3. A small portion (8.6%; 42/491) of the pregnant women reported to get the COVID-19 vaccine if it is recommended by the health authorities and become freely available, whereas majority of them (91.4%; 449/491) showed their refusal to take the COVID-19 vaccine.

**Figure 4:**
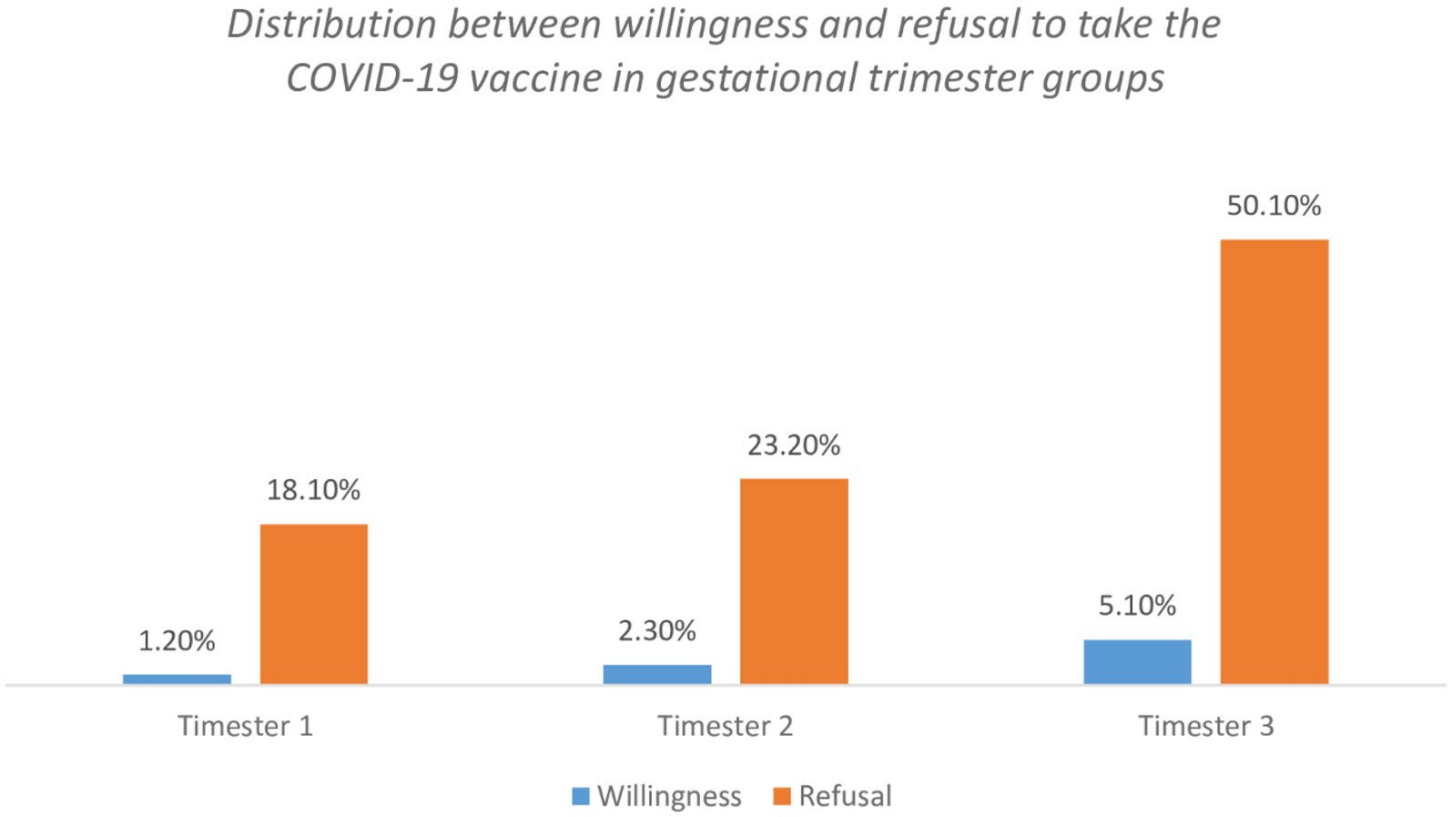
The distribution between willingness and refusal to take the COVID-19 vaccine in gestational trimester groups of pregnant women.

More than half of participants (55.2%; 271/491) were in their third gestational trimester, whereas the rest of them were in their second (25.5%; 125/491) and first trimester (19.3%; 95/491). The refusal rate of taking a COVID-19 vaccine increased gradually with increasing in trimesters, with the third trimester showing the highest refusal rate by pregnant women interviewed (Fig. 4).

To investigate the association of occupation, residence, economic status, education level, husband’s education level, gravidity and parity of pregnant women with willingness to take the COVID-19 vaccine, Chi-square analyses were conducted. Overall, majority of pregnant women (91.4%; 449/491) showed their refusal to take the COVID-19 vaccine. In addition as explained in Table 2 the contained variables did not indicate any significant correlation with willingness to take the COVID-19 vaccine (*P* < 0.05) (Table 2).

**Table 2:** Association between selected socio-demographic variables and COVID-19 vaccine acceptance.

Variable N = 491	Will you take COVID-19 vaccine? (COVID-19 vaccine acceptance)	
	Yes N (%)	No N (%)
**Occupation**		
Employed (184)	16 (8.7)	168 (91.3)
Housewife (307)	26 (8.5)	281 (91.5)
	X^2^ = 0.008 p value = 0.931	
**Residence**		
Urban (369)	32 (8.7)	337 (91.3)
Rural (122)	10 (8.2)	112 (91.8)
	X^2^ = 0.026 p value = 0.871	
**Economic status**		
Good (134)	11 (8.2)	123 (91.8)
Busted (357)	31 (8.7)	326 (91.3)
	X^2^ = 0.028 p value = 0.867	
**Education level**		
Literate (349)	28 (8.0)	321 (92.0)
Illiterate (142)	14 (9.9)	128 (90.1)
	X^2^ = 0.435 p value = 0.510	
**Husband’s Education level**		
Literate (412)	36 (8.7)	376 (91.3)
Illiterate (79)	6 (7.6)	73 (92.4)
	X^2^ = 0.111 p value = 0.739	
**Gravidity**		
Primigravida (189)	17 (9.0)	172 (91.0)
Multigravida (302)	25 (8.3)	277 (91.7)
	X^2^ = 0.076 p value = 0.782	
**Parity**		
Nulliparous (180)	18 (10.0)	162 (90.0)
Multiparous(311)	24 (7.7)	287 (92.3)
	X^2^ = 0.760 p value = 0.383	
Total	42 (8.6%)	349 (91.4%)

Major reasons associated with COVID-19 vaccine refusal were pregnant women’s belief that the COVID-19 vaccine is not safe for their infants (73.4%), I do not need to vaccinate because I have enough immunity (39.3%), the vaccine causes infertility (20.5%), followed by the belief that ‘if I get it, I will die within next two years (20.1%) and ‘It might be low quality for Afghanistan’ (9.6%), as shown in Table 3.

**Table 3:** Reasons for hesitancy to take the COVID-19 vaccine (N = 492).

Item	N (%)	
The vaccine will harm my baby	336	73.4%
I don’t need to vaccine because I have passed the infection and have enough immunity	180	39.3%
The vaccine will cause to infertility	94	20.5%
If I get it, it will die within two next years	92	20.1%
It might be lower quality for Afghanistan	44	9.6%
It has magnetic memories	2	0.4%
Other reason	38	8.3%

**Figure 5:**
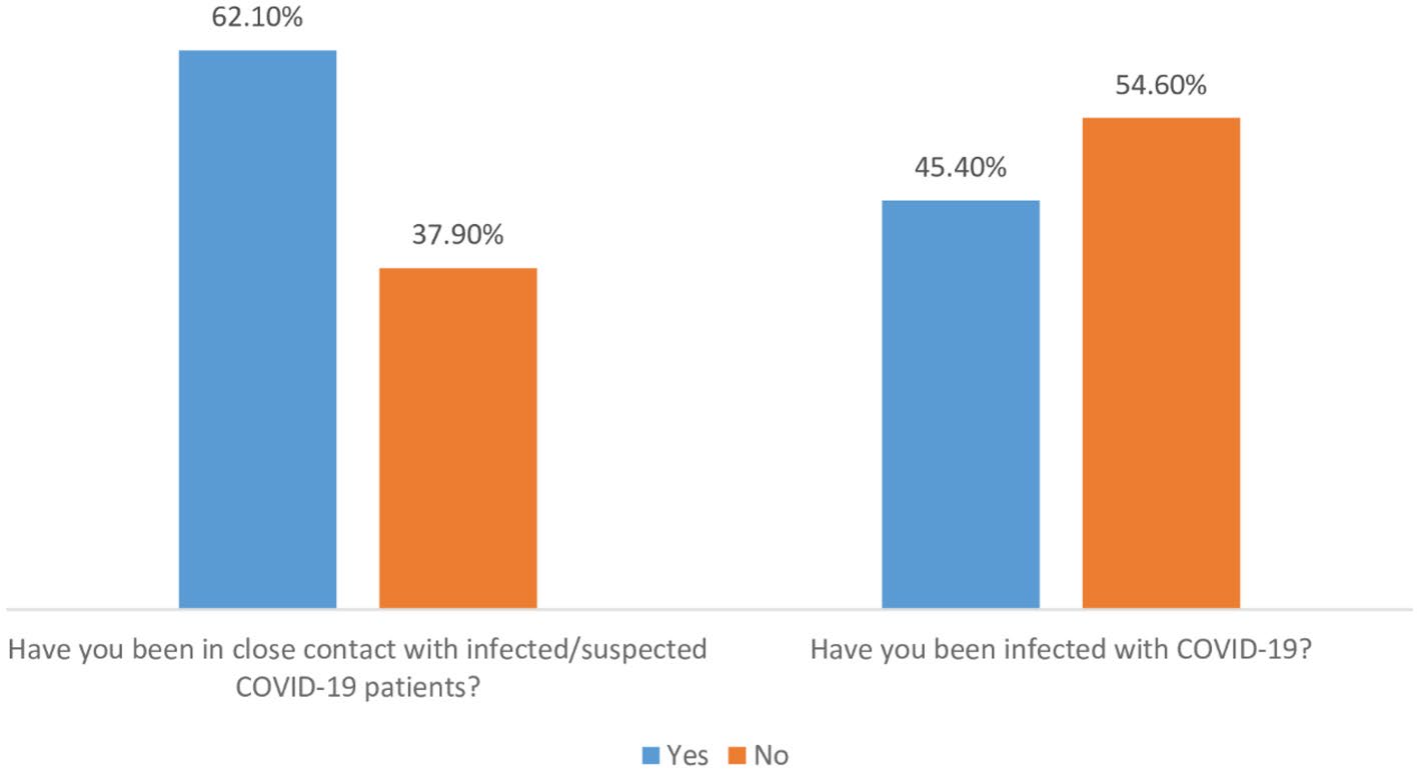
Pregnant women declaration on their close contact with COVID-19 patients and their infection rate by COVID-19.

More than half of pregnant women (62.1%; 305/491) reported that they were in contact with COVID-19 infected/suspected family members (Fig. 5).

## Discussion

Pregnant women with COVID-19 infection are more prone to hospitalization and premature delivery than those without COVID-19, particularly when the infection is accompanied with increased age and underlying chronic diseases. In such circumstance, prevention of infection is a priority for pregnant women. The COVID-19 vaccine is considered as the best choice for women and their infants to prevent the infection, limit the need for intensive care services and minimize the premature birth of babies^7^. Reports suggest that the benefits of COVID-19 vaccination outweigh any known side effects or potential risks of vaccination for pregnant women^8^.

The vaccination of pregnant women to protect the babies from infectious diseases is not a new strategy. Tetanus vaccination is recommended since 30 years to eliminate maternal and neonatal tetanus^26^.

Studies indicated the coverage of full vaccination was low among mothers and children in Afghanistan, especially in remote areas^27,28^. Prolong conflict, lack of health essential knowledge, illiteracy, misinformation, and parental refusal are the key barriers to get the vaccination^29^.

This study evaluated the willingness to get the COVID-19 vaccine in a sample of pregnant women who visited the gynecology wards of several hospitals in Kabul, Afghanistan. To the best of our knowledge, this is the first study among pregnant women toward the COVID-19 vaccine in the country.

The present study found a significantly low acceptance of the COVID-19 vaccine (8.6%; 42/491) among pregnant women. This indicates a lower rate of acceptance as compared to a global survey among pregnant women conducted in 16 countries—the United States, India, Brazil, Russia, Spain, Argentina, Colombia, UK, Mexico, Peru, South Africa, Italy, Chile, Australia, New Zealand and the Philippines (range by country: 28.8–84.4%)^14^. Meanwhile our study finding is significantly lower than what is reported by a multi-center study conducted among pregnant women of China (77.4%)^30^, and what is reported from New York (58.3%)^31^, Ethiopia (70.7%)^32^, and Vietnam (60.4%)^33^.

Pregnant women in the third trimester expressed the highest rate of vaccine refusal as compared to first and second trimesters. This may indicate that pregnant women are more concerned of the adverse effects of the COVID-19 vaccine as they get closer to delivery.

The two most common reasons for hesitancy to take the COVID-19 vaccine were fear of harming the baby (73.4%) and having sufficient immunity due to passing COVID-19 infection (39.3%). The fear of harming infants is also reported by pregnant women in other studies^14,30,32,33^. The second reason was a rare claim reported from pregnant women in our study. The higher rate of infection among public in Afghanistan reported by surveys during the first wave of the pandemic could be a reason that the pregnant women thought they might have sufficient immunity against the COVID-19^34^. Occupation, economic and educational levels of pregnant women and their spouses did not show any correlation with higher refusal rate in our study.

Lastly, television and social media were the main sources of information for pregnant women in our study. These are essential tools to inform all populations about the importance of vaccination.

## Conclusion

The study findings indicated high rates of vaccine refusal among pregnant women in Kabul of Afghanistan, emphasizing the need to reduce the barriers and encourage toward vaccination. This will ensure pregnant women and their infants benefit from vaccine candidates that they will ultimately be protected against COVID-19.

## Data Availability

Data cannot be shared publicly because of ethical restriction and respect for anonymity. Data are available upon request from Dr. Arash Nemat, Academic member of Microbiology Department, Kabul University of Medical Sciences via (dr.arashnemat@yahoo.com).
